# A Comparison of Dietary Protein Digestibility, Based on DIAAS Scoring, in Vegetarian and Non-Vegetarian Athletes

**DOI:** 10.3390/nu11123016

**Published:** 2019-12-10

**Authors:** Corinne Ciuris, Heidi M. Lynch, Christopher Wharton, Carol S. Johnston

**Affiliations:** 1 College of Health Solutions, Nutrition Program, Arizona State University, 550 N. 3rd St., Phoenix, AZ 85004, USA; cczuelke@gmail.com; 2 Kinesiology Department, 3900 Lomaland Dr., Point Loma Nazarene University, San Diego, CA 92106, USA; hlynch@pointloma.edu; 3 College of Health Solutions, Voluntary Radical Simplicity Lab, Arizona State University, 550 N. 3rd St., Phoenix, AZ 85004, USA; christopher.wharton@asu.edu

**Keywords:** vegetarian, protein, lean mass, athlete, endurance, strength

## Abstract

Vegetarian diets provide an abundance of nutrients when carefully planned. However, vegetarian diets may have lower protein quality compared to omnivorous diets, a reflection of less favorable amino acid profiles and bioavailability. Hence, the current recommended dietary allowance for protein may not be adequate for some vegetarian populations. The purpose of this study was to determine dietary protein quality using the DIAAS (Digestible Indispensable Amino Acid Score) method in vegetarian and omnivore endurance athletes. DIAAS scores reflect the true ileal digestibility of the indispensable amino acids that are present in food items, and these scores can be used to compute the available protein in diet plans. Thirty-eight omnivores and 22 vegetarians submitted seven-day food records that were analyzed for nutrient content, and DIAAS scores were computed by diet group. Average available protein (g) was compared along with participants’ lean body mass and strength (quantified using the peak torque of leg extension). DIAAS scores and available protein were higher for omnivorous versus vegetarian athletes (+11% and +43%, respectively, *p* < 0.05). Omnivorous participants had significantly higher lean body mass than vegetarian participants (+14%), and significant correlations existed between available protein and strength (r = 0.314) and available protein and lean body mass (r = 0.541). Based upon available protein, as determined through the DIAAS, vegetarian athletes in this study would need to consume, on average, an additional 10 g protein daily to reach the recommended intake for protein (1.2 g/kg/d). An additional 22 g protein daily would be needed to achieve an intake of 1.4 g/kg/d, the upper end of the recommended intake range.

## 1. Introduction

Approximately 4% of Americans follow a vegetarian diet [1]. Different forms of vegetarianism exist, and some diets may be stricter than others. Pescatarians avoid all meat and poultry besides fish and other seafood, lacto-ovo vegetarians avoid all flesh products, and vegans avoid all animal products including all forms of flesh, dairy and eggs [2]. People may choose not to eat meat for ethical reasons, out of concern for the environment, due to religious beliefs, for potential health benefits, or for a combination of reasons [3,4].

Vegetarian diets can often be more healthy and nutrient-rich than omnivorous diets [5] and, as such, are associated with healthy outcomes, including a lower incidence of heart disease and total cancers, a decreased risk for all-cause mortality, and fewer incidences of metabolic syndrome and diabetes [6,7,8]. These diets may be abundant in fiber and antioxidants, but they may also lack appropriate amounts of iron and other minerals if not planned correctly [9]. The only recommended dietary allowance (RDA) that is different for vegetarians versus omnivores is iron: the Dietary Reference Intakes report, which includes RDAs, suggests that vegetarians should consume 1.8 times more iron than the RDA [10]. This recommendation is crucial because vegetarian sources of iron are less bioavailable. Non-heme iron, generally derived from plant sources, can have an absorption rate as low as 2%, whereas heme iron from animals has absorption rates of about 25% [11]. Other nutrients may also be a cause for concern when avoiding animal foods, and experts recommend the supplementation not only of iron but also of calcium and B-12 for vegans [12,13,14].

Dietary protein may represent similar considerations for plant-based eaters. The current RDA does not differentiate between omnivores and any type of plant-based eater: It remains set at 0.8 g of protein per kg body weight for all adults [15]. This recommendation assumes that plant-based eaters consume at least half of their protein from animal sources [15]; however, this might not be the case for stricter vegetarians and vegans [15]. Some research, for example, has suggested that vegetarians consume as little as 21% of their protein from animal sources [16]. Given the potential for lower animal-based protein intake and higher plant-based protein intake, vegetarians may consume proteins with generally lower digestibility and quality than animal proteins, both of which can be hampered by anti-nutritional factors or an imbalance of amino acids [16]. Additionally, an inadequate intake of specific amino acids may limit protein synthesis in the body [5,17,18]. Specifically, l-leucine appears to play a particularly strong stimulatory role for triggering muscle protein synthesis [19,20], and animal protein typically is higher in leucine than plant protein.

Protein quality and quantity are important issues for athletes in particular, and meeting protein needs can be even more challenging among athletes who are vegetarian or vegan. For example, some recommendations suggest that endurance athletes consume approximately 0.4–0.6 g of protein per kilogram of body weight over the RDA, and athletes participating in sports emphasizing strength and power may require as much as 2.0 g per kilogram of body weight, according to the American College of Sports Medicine and the Academy of Nutrition and Dietetics [21,22]. Endurance athletes also benefit from an increased protein intake compared to the RDA due to their increased protein turnover rates and, potentially, some amino acid oxidation during endurance exercises. Though these recommended levels of protein intake may meet the needs of omnivorous athletes, plant sources may not be as effective as animal sources in contributing to anabolic processes in the body [23]. Research in this area is therefore important for athletes who would like to reduce or eliminate animal products from their diets and still optimize performance.

The purpose of this cross-sectional study was to analyze dietary protein availability using the DIAAS (Digestible Indispensable Amino Acid Score) method and to relate available protein to muscle mass and strength in both vegetarian and omnivorous endurance athletes. DIAAS scores reflect the true ileal digestibility of the indispensable amino acids that are present within individual food items and can be used to compute the available protein in diet plans [24]. It was hypothesized that available dietary protein would be significantly higher among omnivorous athletes compared to vegetarian athletes, that lean body mass (LBM) would be significantly higher in omnivorous athletes compared to vegetarian athletes, that LBM would be significantly correlated to strength, and that available protein would be significantly correlated to LBM and strength.

## 2. Materials and Methods

This cross-sectional study represented a secondary analysis that used data extracted from a study that examined the adequacy of vegetarian diets for supporting sport performance [25]. Participants were competitive endurance athletes (35 vegetarians and 35 omnivores by self-report). Based on responses to questions regarding diet during screening, eight of the vegetarian participants were reclassified as omnivore due to occasional meat consumption. Sixty of the athletes submitted food records, and the data analyses herein represent 22 vegetarians and 38 omnivores. Note, the present sample includes data from three additional omnivore participants, as the original report had 35 omnivore participants (see reference [25]). Participants were healthy by self-report and free of injury. All participants provided written consent, and the study was approved by the Institutional Review Board at Arizona State University (HS1211008557). Recruitment and all data collection occurred between August and November of 2015.

Participants were instructed to record all foods, drinks, and supplements for seven consecutive days. Logs were designed to be as detailed as possible; participants were encouraged to include brand names and portion sizes when applicable. Participants were asked to maintain typical dietary patterns during this recording period. All recorded food items were entered and analyzed using Food Processor 10.11.0 [ESHA Research, Salem, OR, USA] by a dietetics graduate student. A default food list was used during analysis when detail was lacking in the food records or when given items were not available in the Food Processor database.

Data from Food Processor were entered into a DIAAS calculation spreadsheet that encompassed a limited list of food categories derived from the report of the 2011 Food and Agriculture Organization Expert subcommittee [26]. Foods were sorted into the most applicable category using a standard protocol. The DIAAS spreadsheet included several forms of dairy, eggs, meat, grains, beans, legumes, nuts, and seeds. Two types of protein powder were included: whey protein concentrate and soy protein isolate. Fruits, vegetables, sugars, and oils were not included due to their minimal protein contribution. All seven days of food were entered into the DIAAS spreadsheet. Three subjects were missing 1 of 7 daily records, and an additional subject turned in 4 of 7 daily records, but their data remained in the analyses. Four indispensable amino acids, the most common limiting amino acids among plant foods, were included in the calculation spreadsheet: lysine, sulfur amino acids [SAA = methionine + cysteine], threonine, and tryptophan (see Appendix A Table A1 and Reference [24]). All animal flesh foods [meat, poultry, and seafood] were assumed to have 100% digestibility [27] (see Appendix A Table A2). The digestibility score was calculated for daily diets using DIAAS formulas for the common food items, and the 7-day DIAAS average was calculated. Available protein values were calculated by applying digestibility percent to the averaged total protein intake.

Dual-energy X-ray absorptiometry (DEXA; Lunar iDXA, General Electric Company, East Cleveland, OH, USA) was used to determine body fat percentage and LBM. Strength was measured by assessing peak torque on an isokinetic dynamometer (Computer Sports Medicine, Inc., Stoughton, MA, USA) for leg extension, as described previously [25]. Three maximal effort repetitions were performed at three different speeds of 60, 180, and 240 degrees per second, and data are reported in Newton-meters (Nm).

Data are means ± the standard deviation (SD). SPSS 22.0 (SPSS Inc., Chicago, IL, USA) was used to perform all statistical analyses. Data were tested for normal distribution (Kolmogorov–Smirnov test); abnormally distributed data were log-transformed to achieve normal distribution. No outliers were identified (computed as 3 SD from the mean). Pearson correlations were used to evaluate relationships between digestible protein intake, lean body mass, and strength. A univariate analysis was used to evaluate differences between groups. *p* values of <0.05 were considered significant.

## 3. Results

Participants were classified as triathletes (*n* = 27), runners (*n* = 23) or cyclists (*n* = 10). There were no significant differences in strength or LBM between athlete groups (data not shown; see reference [25]). When categorized by diet, age did not vary between vegetarians and omnivores; however, body weight and body mass index (BMI) were significantly higher for omnivores versus vegetarians (Table 1). These differences were retained after controlling for gender (*p* < 0.001). Total energy, total fat, and saturated fat intake did not differ between groups, and carbohydrate intake was significantly higher for vegetarian participants (Table 1). Total protein intake, the DIAAS score, and available protein (g and g/kg) were significantly higher for omnivore participants versus vegetarian participants (+29%, +11%, +43%, and +27%, respectively).

**Figure 1:**
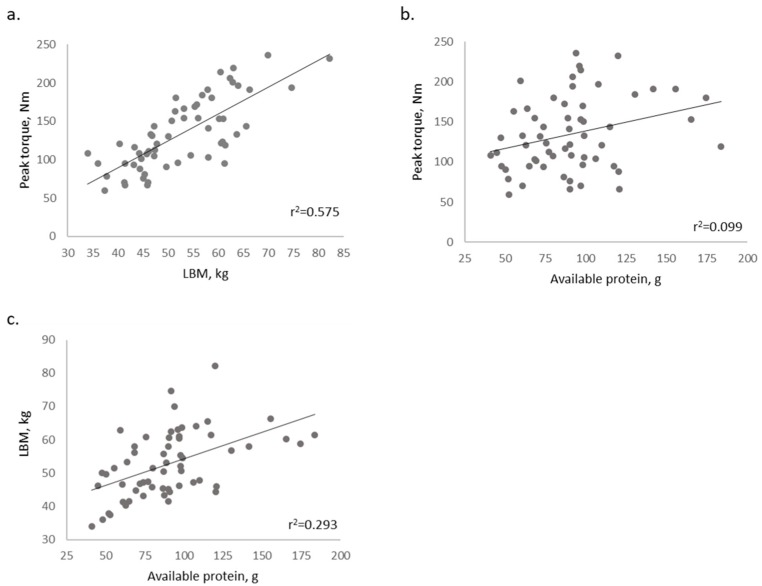
Scatterplots displaying the relationships for (**a**) peak torque (Newton-meters, Nm) and lean body mass (LBM); (**b**) strength and available protein; and (**c**) LBM and available protein. Correlations are significant (*p* < 0.05; r^2^ are depicted for each curve).

LBM differed significantly between the omnivore and vegetarian groups (*p* = 0.011). Though not a statistically significant difference, strength tended to be greater for the omnivore group compared to the vegetarian group (+19%, *p* = 0.074; Table 1). After controlling for gender, the difference in LBM between groups was more distinct (*p* = 0.004); however, the difference in strength between groups was attenuated (*p* = 0.106). Lean body mass and strength were directly correlated (*p* < 0.001), and available protein intake was significantly correlated to both lean body mass (*p* < 0.001) and strength (*p* = 0.016) (Figure 1).

## 4. Discussion

This investigation was designed to evaluate and compare dietary protein adequacy in vegetarian athletes and omnivore athletes by utilizing DIAAS scoring. There was no significant difference in energy intake between groups, but notable differences existed with respect to dietary protein intake. The DIAAS was 11% higher for omnivores than vegetarians (99.9 ± 0.8% and 89.9 ± 10.5%, respectively, *p* < 0.001). The available protein was 43% higher in the diets of omnivorous athletes versus vegetarian athletes, in part a reflection of their greater intake of dietary protein (102 versus 79 g/day). However, when compared to the protein recommendation for competitive athletes (1.2–1.4 g/kg/d), omnivorous athletes appeared to meet the recommendation, based on available protein, (1.4 g/kg/d) while vegetarian athletes fell slightly below the recommended intake range (1.1 g/kg/d). Based on the available protein data, vegetarian athletes in this study would need to consume, on average, an additional 10 g protein daily to reach the low end of the recommended intake for protein (1.2 g/kg/d). An additional 22 g protein daily would be needed to achieve an intake of 1.4 g/kg/d, the upper end of the recommended intake range. These supplementary amounts are comparable to those calculated by others based on the reduced bioavailability of plant-based proteins [16,28]. Such increases in dietary protein (10 or 22 g) could be easily addressed through the addition of a single protein-rich snack or by slightly increasing the amount of protein consumed at each meal/snack throughout the day.

To our knowledge, this is the first report that has used DIAAS calculations to compare the protein digestibility of the diets of free-living vegetarians and omnivores. The DIAAS method was developed in 2013 (see reference [23]) to address the shortcomings of the protein digestibility-corrected amino acid score (PDCAAS) method, which was endorsed by international expert panels and has been used extensively since the early 1990s to assess protein quality for humans [29]. The PDCAAS method utilizes the true fecal digestibility of the entire protein in its calculation, whereas the DIAAS calculation utilizes the ileal digestibility coefficients of each amino acid in a food to determine the true ileal digestibility of the indispensable amino acids present [30]. This is an important distinction, because microbial protein degradation in the large intestine falsely inflates scores using the PDCAAS method. Furthermore, the PDCAAS score is truncated at 1.0, thus attenuating the value of high-quality proteins [30].

The difference in available protein intakes between groups was about 20% (1.1 versus 1.4 g/kg for the vegetarians and omnivores, respectively; *p* = 0.022), and a 16% reduction in strength was noted in the vegetarians in comparison to the omnivores (*p* = 0.074). The significant associations for available protein intake, lean body mass, and strength observed in this study are important findings and may be especially meaningful for vegetarian athlete populations, particularly if having considerable lean body mass and strength are important for their sport. Berryman et al. reviewed 28 studies that examined the role of strength training for improving the performance of endurance athletes beyond that required for sport-specific endurance and concluded that strength training was associated with moderate improvements in performance [31]. This was due largely to improvements in maximal strength and the energy cost of locomotion [31]. In a second systematic review comprising 24 studies, strength training improved running economy by 2–8% [32]. Sufficient available protein may be important for endurance athletes for the maintenance of strength but also due to the increased protein turnover compared to the general population [21]. Our results suggest, however, that only modest increases in protein intake may be needed to aid vegetarian athletes in these outcomes.

The athletes in this study were endurance athletes, and, as such, their sports depend more on their cardiovascular fitness and running or cycling economy than their absolute strength or power. Endurance athletes tend to be very lean, regardless of omnivore or vegetarian status. The vegetarians in this study were significantly lower in total and lean body mass; however, their body fat percentages were not significantly different. In the context of these endurance sports, this is likely not a disadvantage and may be advantageous. For example, the absolute maximal oxygen uptake (VO2max, L/min) between these vegetarian and omnivores did not differ [25]. However, when expressed relative to body weight (mL/kg/min), VO2max was significantly higher among female vegetarians compared to female omnivores. No such effect was observed among males. Had these been athletes whose sports require high levels of strength and power (such as weightlifting, football, wrestling), particularly sports that benefit from higher body masses, it is possible that a long-term vegetarian diet might make total and lean body mass accrual (and, consequently, strength development) more challenging. Future work in this area is warranted, as much of the literature relating to vegetarian diets and athletes involves endurance athletes [32,33,34,35].

Some limitations exist in relation to this study. The sample size was small, and it is not known whether participants were in energy balance the week diet records were kept. Because seven-day food records were used for dietary data collection and analysis, it is possible that errors existed in participants’ self-recorded dietary data. To minimize this possibility, participants were instructed to consume their normal diets during the seven-day period and to be as detailed and accurate as possible as they recorded what they consumed. When food logs lacked detail, the research team employed the use of a standardized food item portion list. Though a lack of detail introduced error, the use of a standard list allowed for a consistency of replacement foods and portions across records. Another similar limitation occurred with use of the Food Processor software, as not all food items in participants’ records were available in the software’s food database for analysis. However, these cases were rare, relating generally to specialty foods like protein bars and protein powders from lesser-known brands. When these issues occurred, researchers used the internet and other resources to identify the nutritional content and ingredients of the specialty foods. Replacement items were then selected from Food Processor that approximately matched the content of the participant-recorded food items. Finally, it is important to note that the DIAAS spreadsheet utilized in this research was not extensive, as it was limited by the availability of true ileal digestibility values that have been derived for foods [26]. Quinoa, for example, was not in the DIAAS spreadsheet and was instead categorized under “wheat pasta,” which was considered to be the closest analog among high-protein grains. The rationale for this action was the higher protein content of wheat when compared to other grains on the spreadsheet. Several other food items required a similar method of categorization. Additionally, fruits and vegetables were not included in the DIAAS spreadsheet due to their low protein content and extremely low protein digestibility. It is reasonable to assume that these foods would not contribute large quantities of amino acids to the DIAAS calculation.

Since only endurance athletes were included in this study, these results are not representative of the general population; further research is needed to compare effects of protein quality on lean mass among generally active adults as well as those who are sedentary. Additional research using athletes in strength and power sports would provide a more comprehensive picture of the effect of protein quality on physical performance. Finally, many athletes, and a number of this study’s participants, regularly consume protein supplements in the form of powders, drinks, and bars. The general population may not be as likely to consume these products with consistency, which could result in a considerable variation in dietary protein quality and quantity among non-athlete vegetarians.

## Figures and Tables

**Table 1:** Participant characteristics, dietary intake, and strength *.

Variable	Vegetarian (22)	Omnivore (38)	*p* Value
Gender (M/F)	11/11	23/15	0.601
Age	36.1 ± 9.0	37.9 ± 9.3	0.474
Height (cm)	169.7 ± 8.1	173.7 ± 8.1	0.060
Weight (kg)	63.7 ± 10.2	74.5 ± 12.2	0.001
Lean body mass (kg)	48.5 ± 8.9	55.1 ± 9.6	0.011
Body Mass Index (kg/m^2^)	22.5 ± 2.9	24.6 ± 3.1	0.011
Energy (kcal)	2472 ± 521	2350 ± 637	0.447
Fat (g)	90.8 ± 25.4	86.8 ± 35.1	0.637
Sat fat (g)	23.3 ± 11.1	27.8 ± 11.0	0.162
Carbohydrates (g)	332.4 ± 70.9	280.8 ± 79.7	0.015
Protein (g)	78.5 ± 17.7	101.6 ± 31.2	0.002
Protein (g/kg)	1.2 ± 0.2	1.4 ± 0.5	0.170
DIAAS (%)	89.9 ± 10.5	99.9 ± 0.8	<0.001
Available protein (g)	71.0 ± 19.6	101.5 ± 31.2	<0.001
Available protein (g/kg)	1.1 ± 0.3	1.4 ± 0.5	0.022
Peak torque (Nm) **	120.0 ± 45.6	142.4 ± 44.7	0.074

* Data are means ± SD; *p* value for univariate test; ** *n* = 21 for vegetarian due to missing data; Nm, Newton-meters.

## Appendix A

**Table A1:** DIAAS calculation table for mixed diet: Example.

	Wt	Pro	Lys	SAA	Thr		Trp		Lys		SAA		Thr	Trp	Protein Content	Lys	SAA	Thr	Trp	
	(g)	(g/100g)	(g/100g)						Ileal Digestibility						(g)	Digestible mg IAA				
	A	B	C	D	E		F		G		H		I	J	AxB/100	(AxB) X CxG	(AxB) X DxH	(AxB) X ExI	(AxB) X FxJ	
Milk	100	3.4	0.312	0.136	0.177		0.055		0.91		0.93		0.92	0.93	3.4	283.92	126.48	162.84	51.15	
Cereal (corn)	50	7.5	0.07	0.24	0.2		0.04		0.66		0.76		0.69	0.42	3.75	23.1	91.2	69	8.4	
Total	150														7.15	307	217.7	231.8	59.6	
	Amino Acids mg/g (total for each AA/total protein)															42.9	30.4	32.4	8.3	
					Reference Pattern (mg/g)											Digestible IAA Reference Ratio				DIAAS for mixture
						Lys		SAA		Thr		Trp				Lys	SAA	Thr	Trp	**89%** **(Lys)**
	Older Children/Adults					48		23		25		6.6				**0.89**	1.32	1.3	1.26	

The above table is adapted from: “Dietary protein quality evaluation in human nutrition.” FAO/WHO 2013. (see ref. [24]).

**Table A2:** Protein information for all foods used in DIAAS calculation spreadsheet.

Food Product	Protein	IAA Composition * (g/100g)				True Ileal IAA Digestibility **			
	g/100g	Lys	SAA	Thr	Trp	Lys	SAA	Thr	Trp
Milk	3.4	0.312	0.136	0.177	0.055	0.91	0.93	0.92	0.93
Egg	12.14	0.82	0.681	0.596	0.194	0.909	0.909	0.909	0.909
Bread	10.5	0.1464	0.2034	0.1536	0.0821	0.92	0.9	0.91	0.9
Cereal (corn)	7.5	0.07	0.24	0.2	0.04	0.66	0.76	0.69	0.42
Cereal (wheat)	11.2	0.3208	0.4126	0.3083	0.1417	0.85	0.79	0.67	0.75
Oats, dry	16.85	0.7012	0.7198	0.575	0.234	0.76	0.85	0.7	0.77
Oats, cooked	2.52	0.135	0.1432	0.0962	0.0402	0.76	0.85	0.7	0.77
Cheese	24.9	2.072	0.777	0.886	0.32	0.91	0.9	0.88	0.85
Cheese, cottage	12.39	1.002	0.49	0.55	0.138	0.91	0.9	0.88	0.85
Corn (flour)	8.5	0.263	0.364	0.351	0.0658	0.76	0.86	0.76	0.78
Tortilla, corn	5.8	0.1625	0.225	0.217	0.0417	0.76	0.86	0.76	0.78
Popcorn	12.86	0.339	0.472	0.454	0.0857	0.76	0.86	0.76	0.78
Beans, cooked	8.21	0.564	0.213	0.346	0.097	0.94	0.77	0.72	0.77
Peas, cooked	8.34	0.602	0.212	0.296	0.093	0.9	0.74	0.91	0.89
Peanuts, dry-roasted	23.68	0.85	0.595	0.811	0.23	0.94	0.96	0.89	0.84
Potato, baked	2.61	0.101	0.0436	0.0601	0.0178	0.52	0.52	0.48	0.47
Sweet potato, baked	2.02	0.0842	0.0648	0.107	0.0404	0.53	0.55	0.51	0.47
Rice, cooked	2.7	0.0968	0.1178	0.0962	0.0312	0.92	0.91	0.82	0.89
Soybean, boiled	16.64	1.108	0.492	0.723	0.242	0.8	0.72	0.81	0.68
Soybean, roasted	35.22	2.344	1.042	1.53	0.512	0.8	0.72	0.81	0.68
Soy milk	3.27	0.131	0.027	0.1082	0.038	0.8	0.72	0.81	0.68
Soy protein isolate	82.14	5.327	2.176	3.137	1.116	0.99	0.98	0.98	0.95
Sunflower seeds	22.78	0.937	0.945	0.928	0.348	0.77	0.81	0.77	0.8
Wheat (flour)	10.3	0.228	0.4024	0.2808	0.1272	0.8	0.88	0.83	0.88
Wheat pasta, cooked	5.7	0.1329	0.1778	0.2057	0.0829	0.8	0.88	0.83	0.88
Whey protein concentrate	83.3	7.321	13.278	5.723	1.441	0.91	0.9	0.89	0.87
Meat (all animal flesh)	25.01	2.125	1.013	1.056	0.292	1	1	1	1

* Total protein and amino acid composition information obtained from Pennington & Spungen ^1^ ** True ileal digestibility values obtained from Gilani et al. ^2^, Evenepoel, et al. ^3^. ^1^ Pennington, J.A.; Spungen, J. *Bowes & Church’s Food Values of Portions Commonly Used*, 19th ed.; Lippincott Williams & Wilkins: Baltimore, MD, USA, 2010. ^2^ Gilani, S.; Tome, D.; Moughan, P.; Burlingame, B. Report of a Sub-Committee of the 2011 FAO Consultation on “Protein Quality Evaluation in Human Nutrition” on: The Assessment of Amino Acid Digestibility in Foods for Humans and including a Collation of Published Ileal Amino Acid Digestibility Data for Human Foods. 2011. Available online: http://www.fao.org/ag/humannutrition/36216-04a2f02ec02eafd4f457dd2c9851b4c45.pdf (accessed on 29 September 2019). ^3^ Evenepoel, P.; Geypens, B.; Luypaerts, A.; Hiele, M.; Ghoos, Y.; Rutgeerts, P. Digestibility of Cooked and Raw Egg Protein in Humans as Assessed by Stable Isotope Techniques. *J. Nutr.* **1998**, *128*, 1716–1722.
